# Adiposity and risks of vascular and non-vascular mortality among Chinese adults with type 2 diabetes: a 10-year prospective study

**DOI:** 10.1136/bmjdrc-2021-002489

**Published:** 2022-01-18

**Authors:** Andri Iona, Fiona Bragg, Yu Guo, Ling Yang, Yiping Chen, Pei Pei, Jun Lv, Canqing Yu, Xiaohuan Wang, Jinyi Zhou, Junshi Chen, Robert Clarke, Liming Li, Sarah Parish, Zhengming Chen, the China Kadoorie Biobank Collaborative Group

**Affiliations:** 1 Clinical Trial Service Unit & Epidemiological Studies Unit (CTSU), Nuffield Department of Population Health, University of Oxford, Oxford, UK; 2 Medical Research Council Population Health Research Unit (MRC PHRU), Nuffield Department of Population Health, University of Oxford, Oxford, UK; 3 Fuwai Hospital Chinese Academy of Medical Sciences, National Center for Cardiovascular Diseases, Beijing, China; 4 Chinese Academy of Medical Sciences, Beijing, China; 5 Department of Epidemiology and Biostatistics, School of Public Health, Peking University Health Science Center, Beijing, China; 6 NCDs Prevention and Control Department, Hainan Centre for Disease Control and Prevention, Haikou, Hainan, China; 7 NCDs Prevention and Control Department, Jiangsu Centre for Disease Control and Prevention, Nanjing, Gulou District, China; 8 China National Center for Food Safety Risk Assessment, Beijing, China

**Keywords:** adiposity, CVD, mortality, diabetes mellitus, type 2

## Abstract

**Introduction:**

Among individuals with diabetes, high adiposity has been associated with lower cardiovascular disease (CVD) mortality (the so-called ‘obesity paradox’ phenomenon) in Western populations, for reasons that are still not fully elucidated. Moreover, little is known about such phenomena in Chinese adults with diabetes among whom very few were obese. We aimed to assess the associations of adiposity with vascular and non-vascular mortality among individuals with diabetes, and compare these with associations among individuals without diabetes.

**Research design and methods:**

In 2004–2008, the prospective China Kadoorie Biobank recruited >512 000 adults from 10 areas in China. After ~10 years of follow-up, 3509 deaths (1431 from CVD) were recorded among 23 842 individuals with diabetes but without prior major diseases at baseline. Cox regression yielded adjusted HRs associating adiposity with mortality.

**Results:**

Among people with diabetes, body mass index (BMI) (mean 25.0 kg/m^2^) was positively log linearly associated with CVD incidence (n=9943; HR=1.19 (95% CI 1.15 to 1.22) per 5 kg/m^2^), but showed U-shaped associations with CVD and overall mortality, with lowest risk at 22.5–24.9 kg/m^2^. At lower BMI, risk of death (n=671) within 28 days of CVD onset was particularly elevated, with an HR of 3.26 (95% CI 2.29 to 4.65) at <18.5 kg/m^2^ relative to 22.5–24.9 kg/m^2^, but no higher mortality risk at BMI ≥25.0 kg/m^2^. These associations were similar in self-reported and screen-detected diabetes, and persisted after extensive attempts to address reverse causality and confounding. Among individuals without diabetes (mean BMI 23.6 kg/m^2^; n=23 305 deaths), there were less extreme excess mortality risks at low BMI.

**Conclusions:**

Among relatively lean Chinese adults with diabetes, there were contrasting associations of adiposity with CVD incidence and with mortality. The high mortality risk at low and high BMI levels highlights, if causal, the importance of maintaining normal weight among people with diabetes.

**Significance of this study**

**What is already known about this subject?**

- Western population studies of individuals with diabetes showed that high adiposity levels are associated with lower cardiovascular and overall mortality risk, leading to the notion of the so-called ‘obesity paradox’.
- Evidence on such associations among the relatively lean Chinese population with diabetes is rather limited.

**What are the new findings?**

- Our study found that among Chinese adults with diabetes, body mass index (BMI) was positively and log linearly associated with incidence of cardiovascular disease.
- There were U-shaped associations of BMI with cardiovascular and overall mortality, with the lowest mortality risk at BMI 22.5–24.9 kg/m^2^ and highest at BMI <18.5 kg/m^2^.
- The excess mortality risk at low BMI was particularly marked for deaths occurring immediately after cardiovascular disease onset (short-term mortality), and persisted after extensive attempts to control for reverse causality and confounding.

**How might these results change the focus of research or clinical practice?**

- The opposing associations of BMI with cardiovascular disease incidence and mortality (particularly short-term mortality) at low BMI suggest it may be important for individuals with diabetes to maintain normal weight.
- Further studies including genetic data may help confirm (or refute) whether the observed excess mortality at low BMI is causal.

## Introduction

Adiposity is a major risk factor for cardiovascular diseases (CVDs).^1 2^ However, the relevance of adiposity, including both the shape and strength of associations, for short-term and long-term mortality risks following incident CVD events remains uncertain.^3^ This is particularly true among individuals with conditions caused by excess adiposity or with potential to result in weight change, such as type 2 diabetes.^4 5^ There is evidence that among individuals with diabetes in Western populations, high adiposity is associated with lower CVD and overall mortality (ie, the so-called ‘obesity paradox’ phenomenon).^6–8^ The factors underlying this phenomenon are incompletely understood, but have been suggested to reflect physiological phenomena (eg, sarcopenia) or non-causal methodological phenomena (eg, existence of comorbidities, reverse causality).^6^ Understanding these associations may help inform appropriate weight management among patients living with diabetes, and is of particular relevance given the escalating prevalence of diabetes worldwide.^9^

Among individuals with diabetes, several large prospective studies have reported U-shaped^7 8^ or reverse J-shaped^6 10 11^ associations of body mass index (BMI) with all-cause mortality across ethnically diverse populations. Similarly, a few large Western population studies have demonstrated U-shaped associations of BMI with CVD mortality,^6–8^ contrasting with the clearly positive association of BMI with CVD incidence throughout the broad BMI range.^12 13^ However, the evidence is more limited in East Asian populations, with a few studies failing to show any clear association of BMI with CVD mortality.^11 14^ It is possible that the excess CVD mortality observed at lower BMI levels might be due to uncontrolled biases (eg, reverse causality), or residual confounding (eg, by smoking), but studies attempting to address these issues (eg, by excluding the first few years of follow-up and/or restricting analyses to never smokers) have reported contradictory results.^6 10 14^ However, no large studies have examined specifically the time course of death following disease onset (eg, deaths within, vs deaths after, the first 28 days), and few studies have directly compared these associations for CVD incidence versus CVD mortality, and between individuals with and without diabetes in the same population.^6 10^ Furthermore, much of the existing evidence is based largely on studies of Western populations.^6–8^ Given known differences in patterns of adiposity between European and East Asian populations, including typically lower mean BMI,^15^ but higher percentage body fat for a given BMI,^16^ and a greater propensity to central adiposity,^17^ in East Asian populations, the generalizability of these findings is unclear. This is of considerable importance given the high prevalence and frequent suboptimal management of diabetes among adults in China.^18 19^

Using data from the China Kadoorie Biobank (CKB) prospective cohort study, the aim of the present report was to comprehensively assess the associations of adiposity with CVD and all-cause mortality, both overall and by time after disease onset, among individuals with diabetes, comparing these with associations among individuals without diabetes.

## Methods

### Study population

Details of the CKB design, methods and participants have been reported previously.^20 21^ Briefly, participants were recruited between 2004 and 2008 from 10 diverse areas (five urban, five rural), chosen from China’s nationally representative Disease Surveillance Points (DSP) to ensure geographic and social diversity, as well as diversity in exposure and disease patterns, and taking account of the quality of death and disease registries, and local commitment and capacity. All 1 801 200 permanent, non-disabled residents aged 35–74 years were identified through local residential records and invited to participate, and 512 869 men and women were enrolled (including 12 665 just outside the target age range, making the actual range of 30–79 years).

### Data collection

Trained health workers administered laptop-based questionnaires at local study clinics collecting information on sociodemographic and lifestyle factors (eg, smoking, alcohol consumption, diet, physical activity) and medical history. Blood pressure, lung function and anthropometric measures (see below) were recorded using standard protocols. A non-fasting venous blood sample was collected (with time since last food recorded) for storage and on-site random plasma glucose (RPG) testing using the SureStep Plus system (LifeScan, Milpitas, California, USA). Participants with an on-site RPG level between 7.8 and 11.0 mmol/L were invited back for a fasting plasma glucose (FPG) test the following day. Resurveys were undertaken in 2008 and 2013/2014, including a randomly selected sample of approximately 5% of surviving participants, and collecting similar information as at baseline with certain enhancements.

### Definition of diabetes at baseline

Self-reported diabetes was defined by a ‘yes’ response to the question, ‘Has a doctor ever told you that you had diabetes?’ Individuals with self-reported diabetes provided information about age at first diagnosis and current use of diabetes and CVD (eg, statins) medications. Among individuals without self-reported diabetes, screen-detected diabetes was defined as an RPG level ≥7.0 mmol/L with time since last food ≥8 hours, or ≥11.1 mmol/L with time since last food <8 hours, or an FPG level ≥7.0 mmol/L on subsequent testing.^22^ Diabetes at baseline was defined as either self-reported or screen-detected diabetes in the baseline survey.

### Anthropometric measures

Anthropometric measurements were recorded with participants wearing light clothing but without shoes, and usually to the nearest 0.1 cm or 0.1 kg. Standing height and sitting height were measured using a stadiometer, and weight and body fat percentage were measured using a body composition analyzer (TANITA-TBF-300GS; Tanita), with subtraction of the weight of clothing (0.5–1.5 kg depending on the season). Waist circumference (WC) was measured using a non-stretchable tape, mid-way between the lowest rib and the iliac crest. Hip circumference was measured at the maximum circumference around the buttocks. BMI was calculated as weight in kilograms divided by the square of the height in meters. Waist-to-hip ratio was calculated as the ratio of WC to hip circumference. Fat body mass was calculated as the product of body fat percentage and weight, and lean body mass was calculated by subtracting fat body mass from weight.

### Follow-up for morbidity and mortality

Information on incident disease events was obtained from linkage to electronic health insurance records (>98% coverage), supplemented, for certain diseases (stroke, ischemic heart disease (IHD), diabetes, and cancer), by linkages to available disease registries. Mortality was monitored through the Chinese Center for Disease Control and Prevention’s DSP system, with annual checks against local residential and administrative records. For the few deaths without recent medical attention, standardized verbal autopsy procedures were used to determine probable cause of death. Underlying causes of death and disease were coded according to the International Statistical Classification of Diseases and Related Health Problems, Tenth Revision, by trained staff blinded to baseline information. The main outcomes examined were incident CVD and all-cause, CVD and non-CVD mortality, and their major components (online supplemental eTable 1). Non-fatal CVD was defined as a first-ever CVD event without death within 28 days of onset of the event. Fatal CVD was defined as all other first-ever CVD events (ie, a first-ever CVD event with death from any cause within the following 28 days).

10.1136/bmjdrc-2021-002489.supp1 Supplementary data

### Statistical analysis

The present study excluded individuals with self-reported, doctor-diagnosed major diseases at baseline, including stroke/transient ischemic attack (n=8884), IHD (n=15 472), cancer (n=2577), chronic obstructive pulmonary disease (COPD; n=37 055) and tuberculosis (n=7659), and those with missing or implausible values of anthropometric measurements (n=240). After these exclusions, 446 713 remained in the present analyses.

All analyses were performed separately for participants with and without diabetes at baseline. The prevalence or mean values of baseline characteristics were calculated across four BMI categories (cut-points: 18.5, 25.0 and 30.0 kg/m^2^), standardized to the age (5-year groups), sex and study area structure of the study population. Cox proportional hazards models, with time since baseline as the timescale, were used to estimate HRs for the specified disease outcomes, stratified by age at risk (5-year age groups), sex, and study area (ten groups), and adjusted for education (no formal education, primary school, middle school, high school, college/university), smoking (never, occasional, ex-regular, current regular), alcohol intake (never, occasional intake, ex-regular, reduced intake, weekly intake) and physical activity (metabolic equivalent of task hours/day).^23^ To ensure reasonable numbers of participants in each group, adiposity measures were categorized into five groups for BMI (<18.5, 18.5–22.4, 22.5–24.9 (reference group), 25.0–29.9 and ≥30.0 kg/m^2^) and into sex-specific quintiles for other measures (and for BMI when comparing associations of different adiposity measures with disease outcomes). The floating absolute risk method was used to estimate group-specific 95% CIs for each log HR to enable comparisons between any two categories (rather than just pairwise comparisons with the reference category).^24^

The main associations were additionally examined excluding individuals who died during the first 5 years of follow-up (which showed no strong evidence of departure from the proportional hazards assumption) and, separately, ever-regular smokers, across strata of certain baseline characteristics (eg, sex, study area, age, diabetes medication use), and separately among individuals with self-reported and screen-detected diabetes. Sensitivity analyses further excluded individuals with poor self-rated health, other self-reported prior diseases at baseline, and diseases developed during follow-up, and additionally adjusted for RPG, systolic blood pressure (SBP) and heart rate.

All analyses used SAS V.9.3. Figures were produced using R V.3.3.2.

## Results

### Participant characteristics

By 1 January 2016, a total of 44 066 (9%) had died, and 4751 (<1%) were lost to follow-up in the full CKB cohort. Of the 446 713 participants included in the analyses, 23 842 (5.3%) had diabetes at baseline, with a similar prevalence of self-reported (n=11 995, 2.9%) and screen-detected (n=11 847, 2.7%) diabetes. Compared with those without diabetes, individuals with diabetes had significantly higher mean (SD) BMI (25.0 (3.4) vs 23.6 (3.1) kg/m^2^, p<0.0001) and prevalence of overweight (BMI 25.0–29.9 kg/m^2^, 40.8% vs 28.3%) and obesity (BMI ≥30.0 kg/m^2^, 8.3% vs 3.7%). Participants with diabetes tended to be older and less physically active, and to have higher prevalence of hypertension and poor self-rated health than those without diabetes (table 1). BMI was strongly positively associated with SBP. The prevalence of poor self-rated health was highest among underweight (BMI <18.5 kg/m^2^) individuals.

**Table 1:** Characteristics of participants with and without diabetes by BMI at baseline

Characteristics	No diabetes, BMI (kg/m^2^)					Diabetes, BMI (kg/m^2^)				
	<18.5 (n=16 947)	18.5 to <25.0 (n=270 403)	25.0 to <30.0 (n=119 736)	≥30.0 (n=15 785)	All (n=422 871)	<18.5 (n=527)	18.5 to <25.0 (n=11 613)	25.0 to <30.0 (n=9719)	≥30.0 (n=1983)	All (n=23 842)
Mean BMI (SD), kg/m^2^	17.6 (0.9)	22.1 (1.7)	26.8 (1.4)	31.7 (2.1)	23.6 (3.1)	17.5 (0.7)	22.6 (1.6)	27.0 (1.4)	32.0 (2.1)	25.0 (3.4)
Age and socioeconomic factors										
Age, years (SD)	53.0 (14.2)	50.2 (10.3)	50.8 (10.0)	50.6 (11.9)	50.6 (10.2)	58.2 (14.8)	57.4 (9.7)	56.6 (9.4)	55.2 (11.0)	57.0 (9.4)
Women (%)	61.7	58.7	60.9	71.2	59.8	59	60.5	61.7	70.2	61.9
Urban (%)	32.8	39.1	50.4	55.8	42.6	32.7	54.2	64.2	69.5	59
≥6 years of education (%)	50.1	51	50.9	49	50.6	48.3	47.9	46.4	44.7	46.8
Lifestyle factors										
Ever-regular smoker (%)										
Men	79.4	75.3	70.7	71	74.2	79.6	72.2	69.6	72.6	71.4
Women	4.3	2.6	2.3	2.8	2.6	9.7	4.1	4.1	5.1	4.2
Regular alcohol consumption (%)										
Men	32.1	37.3	37.2	35.9	37.2	38.6	37.9	37.1	39.8	37.6
Women	2.4	2.4	2.5	2.1	2.4	3.5	2	1.8	1.8	1.9
Physical activity (SD), MET-hours/day	21.9 (15.8)	22.5 (11.9)	21.4 (12.4)	20.1 (15.1)	22.1 (11.9)	15.7 (11.2)	16.7 (10.4)	16.1 (10.3)	15.7 (11.6)	16.4 (10.1)
Anthropometry, blood pressure and plasma glucose, mean (SD)										
Weight (kg)	44.5 (4.9)	55.9 (5.8)	67.8 (6.0)	80.3 (9.0)	59.7 (9.0)	43.5 (3.8)	56.7 (5.9)	68.0 (5.8)	80.9 (8.0)	63.0 (9.7)
Standing height (cm)	159 (7.7)	159 (5.5)	159 (5.7)	159 (7.1)	159 (5.4)	157 (6.4)	158 (5.7)	159 (5.5)	159 (5.9)	158 (5.4)
Waist circumference (cm)	66 (6.4)	76 (6.3)	88 (6.2)	99 (8.7)	80 (8.9)	67 (5.4)	80 (6.4)	90 (6.0)	101 (7.5)	86 (9.3)
Waist-to-hip ratio	0.80 (0.08)	0.86 (0.06)	0.92 (0.06)	0.96 (0.07)	0.88 (0.06)	0.82 (0.07)	0.90 (0.06)	0.94 (0.06)	0.97 (0.07)	0.92 (0.07)
Percentage body fat	16.8 (3.6)	25.5 (4.4)	33.6 (4.6)	40.1 (7.0)	28.0 (6.4)	16.7 (3.1)	26.7 (4.5)	34.1 (4.5)	40.8 (6.6)	30.7 (6.7)
Fat body mass (kg)	7.4 (2.0)	14.2 (3.5)	22.6 (4.1)	32.0 (7.3)	17.0 (6.1)	7.2 (1.6)	15.1 (3.6)	23.0 (4.0)	32.7 (6.7)	19.6 (6.8)
Lean body mass (kg)	37.2 (4.2)	41.7 (3.8)	45.2 (4.5)	48.3 (7.2)	42.7 (4.5)	36.3 (3.2)	41.6 (3.9)	45.1 (4.4)	48.2 (6.6)	43.4 (4.8)
SBP (mm Hg)	120 (25)	127 (19)	135 (20)	142 (26)	130 (19)	126 (23)	138 (22)	144 (21)	148 (24)	141 (22)
Heart rate (bpm)	80 (16)	78 (11)	79 (12)	81 (16)	79 (11)	87 (15)	83 (13)	83 (13)	84 (16)	83 (12)
Random plasma glucose* (mmol/L)	5.6 (1.5)	5.6 (1.1)	5.8 (1.2)	6.0 (1.7)	5.7 (1.1)	13.8 (7.1)	12.5 (6.0)	12.1 (5.2)	11.9 (6.2)	12.3 (5.5)
Medical history (%)										
Hypertension†	2.8	6.6	13.6	21.8	8.9	8.8	18.9	29.9	38.2	24.5
Chronic liver disease†	1.4	1.2	1.1	1.1	1.2	1.1	1.4	1.1	1.2	1.3
Chronic kidney disease†	1.3	1.3	1.5	1.6	1.3	1.2	1.6	1.8	2.2	1.7
Self-rated poor health	14.4	7.8	7.4	10.2	8	29.9	18	15.7	17.5	17.1

Adjusted for age, sex and study area, as appropriate.

*In a subset of 439 628 participants.

†Self-reported doctor diagnosed.

BMI, body mass index; MET, metabolic equivalent of task; SBP, systolic blood pressure.

### Association of BMI with CVD incidence and mortality

**Figure 1:**
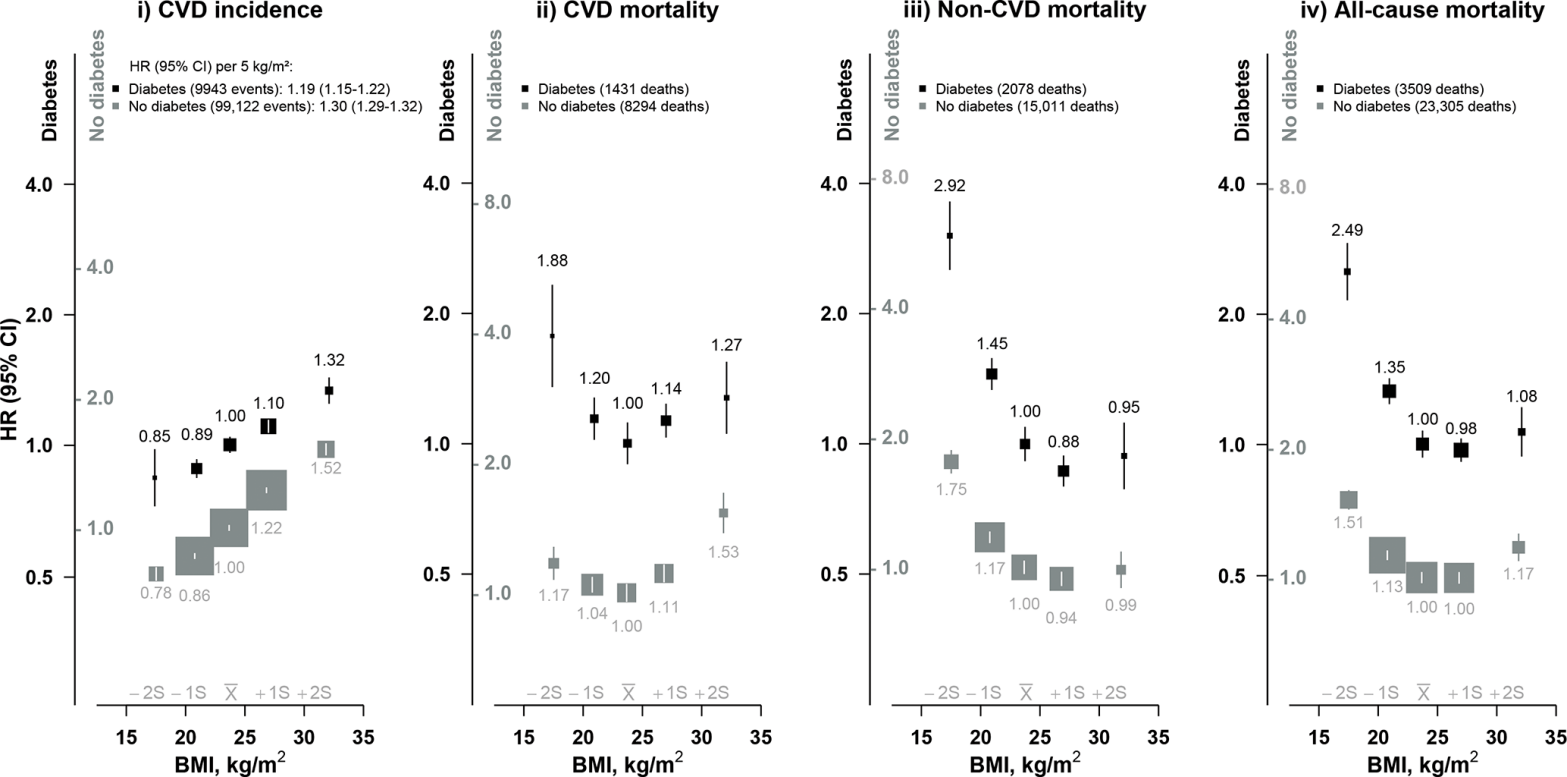
Association of body mass index (BMI) with cardiovascular disease (CVD) incidence and CVD, non-CVD and all-cause mortality. HRs are stratified by age at risk, sex and study area, and adjusted for education, smoking, alcohol and physical activity. HRs are plotted on a floating absolute risk scale and separate y-axis scales were used for individuals with and without diabetes (black and gray labels, respectively). HRs are relative to 22.5–24.9 kg/m^2^ group, separately in individuals with and without diabetes. Each closed square represents HR with the area inversely proportional to the variance of the log HR. Vertical lines indicate 95% CIs. The x̅ above the x-axis represents the mean value of BMI in the full China Kadoorie Biobank population and the ±1S and ±2S represent 1 and 2 SD from the mean, respectively. The p value for trend test for CVD incidence regardless of diabetes status is <0.0001. The p value for trend test at BMI <25 kg/m^2^ for CVD mortality among individuals with diabetes is <0.0001 and among individuals without diabetes is 0.0004. The p value for trend test at BMI <25 kg/m^2^ for non-CVD and all-cause mortality outcomes regardless of diabetes status is <0.0001.

During about 10 years of follow-up, 3509 participants with diabetes died between ages 35 and 79 years (online supplemental eTable 2), and 9943 experienced a first-ever CVD event (3741 IHD, 4501 stroke). The CVD incidence was 55.5 per 1000 person-years among individuals with diabetes and 26.3 per 1000 person-years among individuals without diabetes. There was a strong positive and log-linear association of BMI with CVD incidence throughout the wide adiposity range examined, with each 5 kg/m^2^ higher BMI associated with an adjusted HR of 1.19 (95% CI 1.15 to 1.22) (figure 1). In contrast, BMI showed a U-shaped association with CVD mortality; the risk was lowest among individuals with BMI 22.5–24.9 kg/m^2^, and was approximately twofold higher among those with BMI <18.5 kg/m^2^ (1.88, 95% CI 1.43 to 2.45), more extreme than the excess risk at BMI 25.0–29.9 kg/m^2^ (1.14, 95% CI 1.04 to 1.24) or ≥30.0 kg/m^2^ (1.27, 95% CI 1.07 to 1.54). Largely similar associations were observed with mortality from different types of CVD (ie, IHD, total stroke, ischemic stroke), but with a somewhat smaller excess mortality from intracerebral hemorrhage at low BMI (online supplemental eFigure 1). There were reverse J-shaped associations of BMI with non-CVD and all-cause mortality. Underweight was associated with an approximately threefold higher risk of non-CVD mortality (2.92, 95% CI 2.44 to 3.51) compared with BMI 22.5–24.9 kg/m^2^, with no significant excess risk among those who were overweight or obese. A similar reverse J-shaped association was seen for different non-CVD causes of death, including COPD, cancer, diabetic ketoacidosis and coma, and chronic kidney disease (online supplemental eFigure 2).

### Association of BMI with immediate and long-term mortality after a non-fatal CVD event

**Figure 2:**
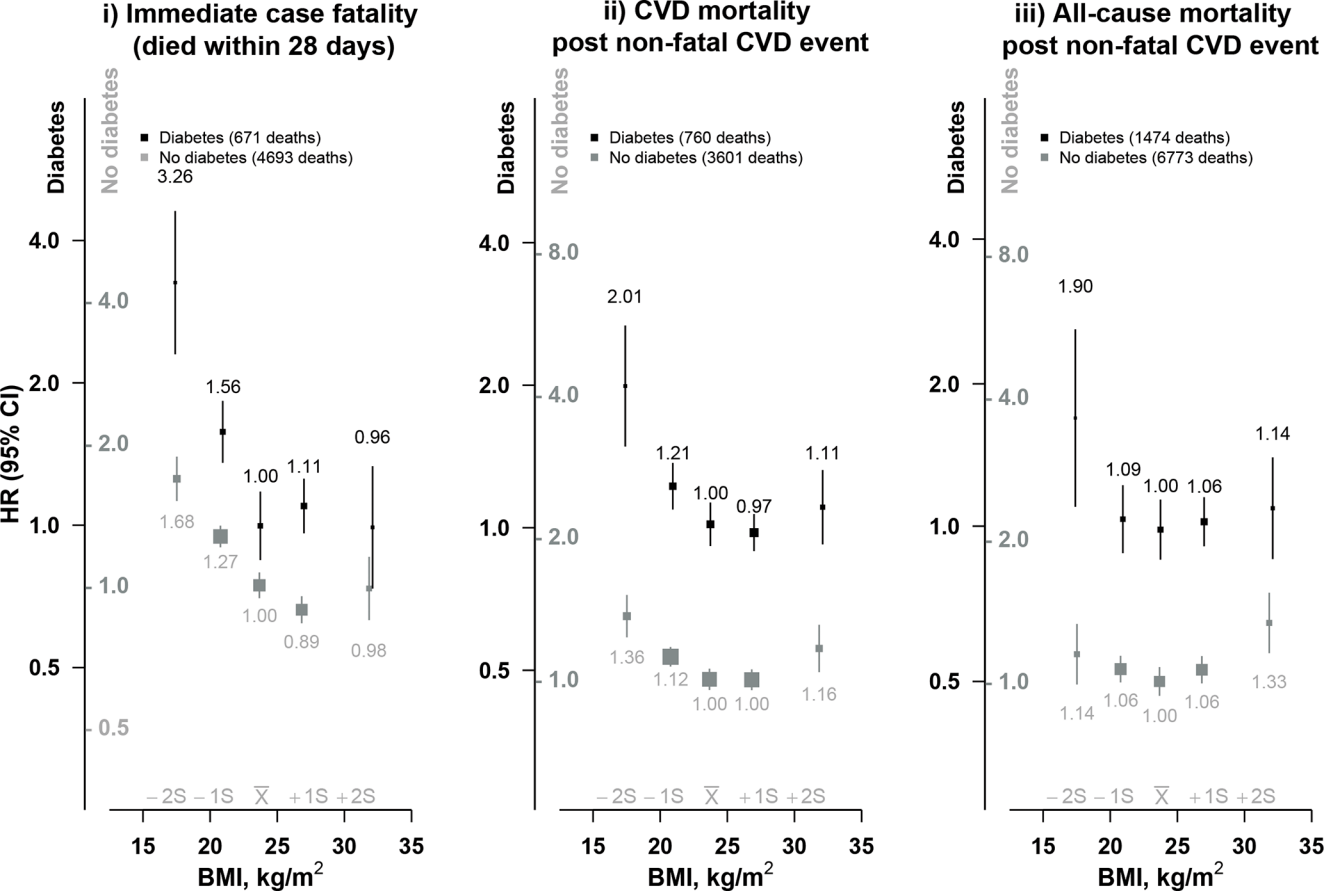
Association of body mass index (BMI) with immediate and long-term mortality after a cardiovascular disease (CVD) event. Conventions as figure 1. Immediate case fatality: mortality from any cause within 28 days from the first CVD event. Non-fatal CVD event: first CVD event not followed by death within 28 days.

When deaths at different time points following onset of a CVD event were considered separately, the excess risk at BMI <25 kg/m^2^ was slightly stronger for immediate deaths (ie, case fatality) than for late CVD deaths (ie, deaths after a non-fatal CVD event) (figure 2). The excess early and late mortality at BMI <25 kg/m^2^, contrasting with the positive log-linear association with incident CVD, persisted after analyses were restricted to never-regular smokers, and after excluding the first 5 years of follow-up (online supplemental eFigure 3). Further exclusion of individuals with additional prior self-reported diseases at baseline (hypertension, chronic kidney disease, chronic liver disease), poor self-rated health at baseline, or hospitalization for major chronic diseases during follow-up did not materially alter these associations (online supplemental eFigure 4). Likewise, the associations were similar among individuals with self-reported and screen-detected diabetes (online supplemental eFigure 5). Moreover, among those with self-reported diabetes, the associations differed little across subgroups defined by diabetes medication use (data not shown).

Among individuals without diabetes, the positive log-linear association of BMI with CVD incidence was stronger (HR 1.30 (95% CI 1.29 to 1.32) per 5 kg/m^2^) than that observed among individuals with diabetes (figure 1). Although the shape of the associations of BMI with CVD, non-CVD and all-cause mortality was largely similar to those observed among individuals with diabetes, the excess risks at low BMI were less extreme in the absence of diabetes.

### Subgroup analyses

The associations of BMI with CVD and non-CVD mortality among individuals with diabetes did not differ by sex, and were similar between age groups (<65 years vs ≥65 years) (online supplemental eFigures 6 and 7). Comparison of urban and rural areas indicated that the association between BMI and CVD mortality was reverse J shaped in rural areas and U shaped in urban areas, while for non-CVD mortality, there was a reverse J-shaped association with BMI in both urban and rural areas (online supplemental eFigure 8). Additional adjustment for baseline SBP attenuated the associations of BMI with CVD mortality at BMI ≥30.0 kg/m^2^ (HR from 2.82 (95% CI 2.35 to 3.39) to 2.10 (95% CI 1.75 to 2.53)), while the risk among underweight individuals became slightly more extreme (HR from 3.92 (95% CI 3.02 to 5.10) to 4.16 (95% CI 3.20 to 5.41); data not shown). Additional adjustment for RPG or heart rate did not materially alter the associations (data not shown). Similarly, the shape of the association of BMI with CVD incidence and immediate case fatality and all-cause mortality after a non-fatal CVD event did not alter when sex-specific BMI quintiles were used (online supplemental eFigure 9).

### Associations of other adiposity measures with CVD incidence and mortality

**Figure 3:**
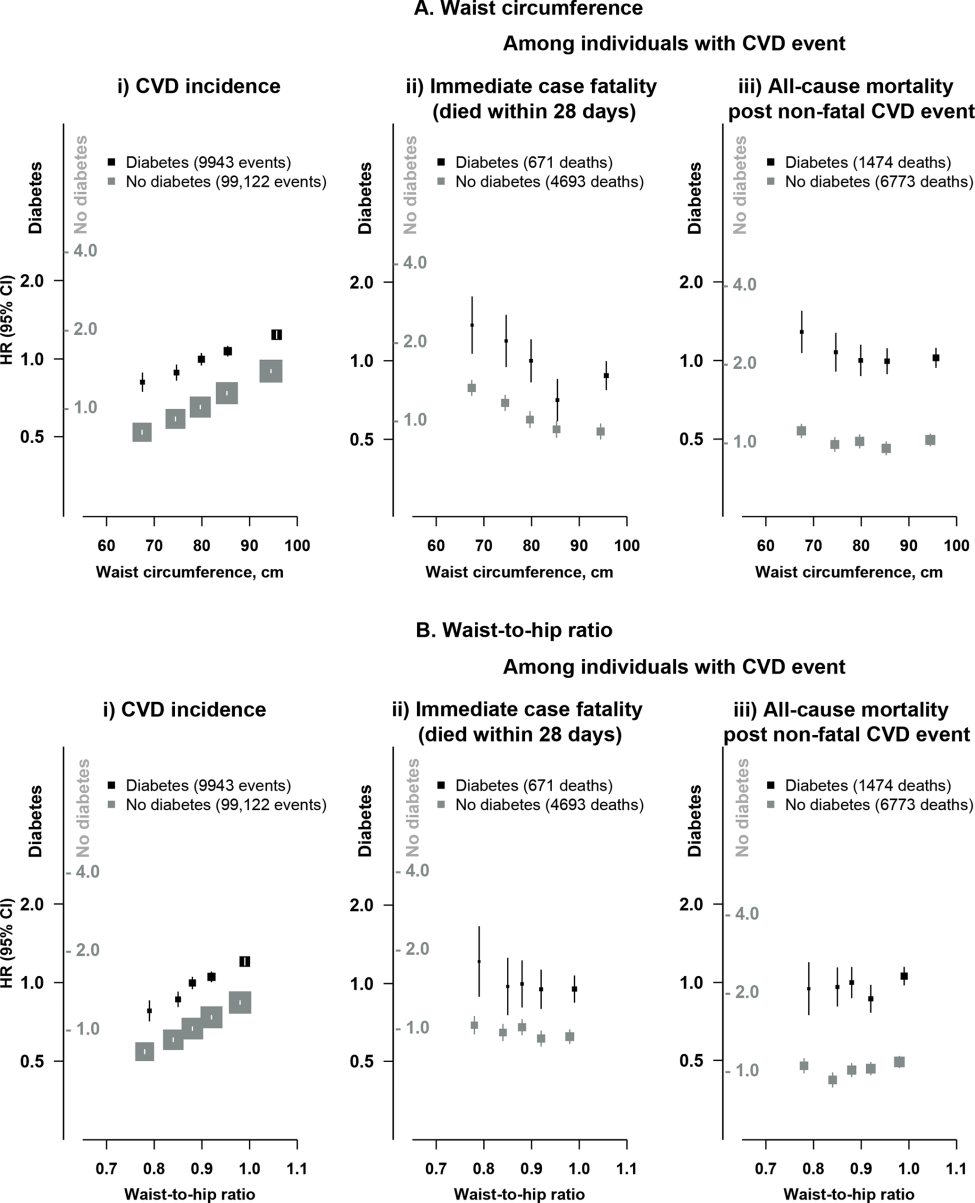
Association of central adiposity with cardiovascular disease (CVD) incidence and immediate and long-term mortality after a CVD event. Conventions as figure 1. HR (95% CI) relative to third quintile group of (A) waist circumference and (B) waist-to-hip ratio, separately in individuals with and without diabetes.

There were strong log-linear positive associations of WC (1.15 (95% CI 1.13 to 1.18) per 1 SD (9.62 cm) higher) and waist-to-hip ratio (1.12 (95% CI 1.10 to 1.14) per 1 SD (0.07) higher) with risk of CVD incidence among individuals with diabetes (figure 3). The associations of adiposity measures other than BMI (WC, waist-to-hip ratio, body fat percentage, lean body mass and fat body mass) with fatal CVD and all-cause mortality after a non-fatal CVD event tended to be flatter than those of BMI, particularly at lower levels of adiposity (online supplemental eFigure 10).

## Discussion

In this large prospective study of relatively lean Chinese adults, adiposity was strongly positively associated with CVD incidence among people with diabetes, with no evidence of any threshold below which lower BMI was no longer associated with lower CVD incidence, at least down to ~18.0 kg/m^2^. In contrast, there was an apparent U-shaped association with CVD mortality, with the lowest risk at BMI levels of 22.5–24.9 kg/m^2^. The risk of immediate case fatality following CVD onset was particularly marked at low BMI, with little excess risk at high BMI levels (≥25.0 kg/m^2^). These associations were similar for different CVD types (eg, IHD and stroke) and adiposity measures (eg, WC), and in previously diagnosed and screen-detected diabetes, and were little altered after extensive attempts to control for reverse causality and confounding. Among individuals without diabetes, there was a similar, although stronger, positive association of BMI with CVD incidence but less extreme excess mortality risk at low BMI.

Most previous prospective studies of adiposity and mortality in diabetes, or meta-analyses of such studies, focused chiefly on all-cause mortality and reported reverse J-shaped associations (with either a null association or small excess mortality risk at high BMI levels),^6 10 11^ as found in the present study, or U-shaped associations.^7 8^ BMI levels associated with the lowest all-cause mortality risk varied across studies, from 22.5 to 24.9 kg/m^2^ in a meta-analysis of 16 studies from diverse populations,^7^ similar to CKB, to 25.0–35.0 kg/m^2^, as in most Western population studies.^6 10^ These findings from the present study, and most previous observational studies among individuals with diabetes, are consistent with more limited data from Mendelian randomization analyses in general Western population studies which reported J-shaped associations between genetically derived BMI and risk of all-cause mortality.^25 26^ However, these genetic studies did not examine the time course of the death. Moreover, all-cause mortality reflected a mixture of causes of death and, as in CKB, over 60% of deaths in these previous studies were due to non-CVD causes.^11 14^ Even for CVD, the relative proportion of different types of CVD (eg, IHD vs stroke) and their associations with adiposity (eg, ischemic vs hemorrhagic stroke^27^) may differ importantly between populations.

Previous evidence on the associations of BMI with CVD mortality in diabetes is limited in East Asian populations, where studies have failed to show any clear association (possibly reflecting small numbers of deaths)^11 14^ and where the mean BMI levels are much lower than in Western populations. The present study now provides robust new evidence of a U-shaped association, consistent with previous large Western population studies,^6–8^ although with the lowest CVD mortality risk at lower BMI levels in Chinese populations. For example, a meta-analysis of 16 prospective, predominantly Western population studies, including ~450 000 individuals with diabetes, reported the lowest CVD mortality risk at BMI 28.0–30.0 kg/m^2^,^7^ much higher than the levels seen in the present study (22.5–24.9 kg/m^2^). These differences in the apparent optimal BMI between populations may reflect differences in patterns and mean levels of adiposity, in the relative burden of different CVD types, or in the completeness of control for biases or confounding.

The present study is the first among individuals with diabetes to examine the associations of adiposity with CVD incidence and mortality simultaneously, including time course of death following CVD onset. BMI was positively log linearly associated with CVD incidence (likely in part reflecting the causal effect on CVD risk of higher blood pressure associated with higher BMI^27^). However, for CVD mortality, especially immediate case fatality, there was a significant excess risk at lower BMI. As in CKB, previous studies have investigated the likely influence of reverse causality (eg, from existing diagnosed or undiagnosed disease at the time of adiposity assessment), and showed that the BMI–mortality association persisted after exclusion of the first few years of follow-up.^6–8 10 11 14^ However, strategies to reduce residual confounding from smoking have shown conflicting findings.^11 14^ A UK Biobank study reported that smoking appeared to act as an effect modifier, with more pronounced excess mortality at low BMI levels among ever smokers than never smokers,^6^ but such an effect was not reported in other studies.^7 10^ Such strategies were extremely limited in studies focused on CVD mortality. Application of these and additional approaches (including exclusion of individuals who developed major diseases during follow-up or with poor self-rated health) in the present study had little effect on the association of BMI with both all-cause and CVD mortality, and the excess mortality risk at low BMI persisted, suggesting that these biases may not have been fully controlled. In CKB, bioimpedance was used to estimate lean and fat body masses, which both demonstrated shallow reverse J-shaped associations with mortality, suggesting that both low lean mass and low fat mass are associated with higher mortality risk. Further detailed investigations in large diabetes populations with more accurate (eg, imaging-derived) measures of lean and fat mass and genetic data are needed to better assess this phenomenon, including the likely causality of the association.

The present study is one of few to compare the associations of BMI with all-cause^6 10^ and CVD^6^ mortality among individuals with and without diabetes. Observed higher cardiovascular risks among individuals with diabetes likely reflect multiple factors, including coexisting hypertension, dyslipidemia and factors associated with insulin resistance.^28 29^ The associations of adiposity with mortality were similar irrespective of diabetes status, but with more extreme mortality risk in diabetes at BMI <22.5 kg/m^2^, similar to UK Biobank findings.^6^ These greater excess mortality risks in diabetes may reflect suboptimal diabetes management resulting in weight, and possibly lean mass, loss, different characteristics of individuals who develop diabetes in the absence of overweight/obesity (eg, greater genetic predisposition),^30^ or the effect of insufficiently controlled biases. However, while previous studies have found that the lowest mortality risk was at higher BMI levels among individuals with, than without, diabetes,^6 10^ the present study found no such relationship.

Our study has several strengths, including the large study population, availability of information on a uniquely wide range of reliably measured adiposity indicators and of cause-specific mortality and incident disease outcomes, and completeness of follow-up. Moreover, the relatively lean population enabled reliable investigations at lower adiposity levels. Furthermore, the associations of adiposity with mortality were examined among individuals both with and without diabetes and, in the former group, separately among those with self-reported and screen-detected diabetes. Finally, the study included comprehensive attempts to control for biases. However, there are also several limitations. First, reverse causality may not have been fully excluded. For example, current data reasonably permit exclusion only of the first 5 years of follow-up, and incorporation of potentially important health states (eg, frailty) and exclusion of individuals with relevant diseases associated with weight change (eg, weight loss associated with the preclinical phase of dementia) are likely incomplete. In addition, further investigations on the genetic associations of BMI with mortality, which are less prone to biases from reverse causality and confounding, are needed to further clarify the nature of these associations. Second, the analyses were based on single adiposity measurements. However, based on repeat measurements among a subset of ~17 000 participants included in the 2008 resurvey (on average 3.6 years after baseline) and ~20 000 included in the 2013/2014 resurvey (7.2 years after baseline) who met the inclusion criteria for the current analyses, the self-correlation coefficient of BMI was ~0.9 (0.93 in the first resurvey and 0.87 in the second resurvey, and similar among those with or without diabetes), suggesting any impact of changes in adiposity was small. Furthermore, accounting for changes in weight during follow-up could increase susceptibility to reverse causality. Finally, collider bias could have influenced the associations of adiposity with mortality among individuals with diabetes,^31–33^ since the risk of developing diabetes is greater among individuals with excess adiposity^4 5^ and is influenced by risk factors for both adiposity and mortality (eg, smoking).^34^

In summary, the present large prospective study of Chinese adults with diabetes (but without known major diseases at baseline) demonstrated opposing associations of BMI with CVD incidence and mortality. Higher BMI was continuously and positively associated with risk of incident, particularly non-fatal, CVD, with the lowest risk observed for CVD incidence at BMI levels <18 kg/m^2^, similar to that observed in individuals without diabetes in CKB and other studies.^35^ For CVD mortality, however, there was a U-shaped association, with BMI levels of 22.5–24.9 kg/m^2^ associated with the lowest mortality risk and those with BMI <18.5 kg/m^2^ having particularly elevated risk of death immediately after CVD onset. Although our study did not provide any clear evidence supporting the notion of the so-called ‘obesity paradox’, the high mortality risk immediately following CVD onset at low BMI, in combination with the high long-term risk of CVD at high BMI levels, highlights the importance of maintaining BMI levels within the so-called ‘normal’ range among people with diabetes.

## Data Availability

Data are available upon reasonable request. The China Kadoorie Biobank (CKB) is a global resource for the investigation of lifestyle, environmental, blood biochemical and genetic factors as determinants of common diseases. The CKB study group is committed to making the cohort data available to the scientific community in China, the UK and worldwide to advance knowledge about the causes, prevention and treatment of disease. For detailed information on what data are currently available to open access users and how to apply for it, visit: http://www.ckbiobank.org/site/Data+Access. Researchers who are interested in obtaining the raw data from the CKB study that underlies this paper should contact ckbaccess@ndph.ox.ac.uk. A research proposal will be requested to ensure that any analysis is performed by bona fide researchers and—where data are not currently available to open access researchers—is restricted to the topic covered in this paper.
