# Supplementary material for: Adiposity and risks of vascular and non-vascular mortality among Chinese adults with type 2 diabetes: a 10-year prospective study
Source: BMJ Open Diabetes Res Care. 2022 Jan 18;10(1):e002489. doi: 10.1136/bmjdrc-2021-002489 (PMC8768914; doi:10.1136/bmjdrc-2021-002489)
Supplement: Supplementary data [file bmjdrc-2021-002489supp001.pdf]

**This page is intentionally left blank**

**Supplementary Material Content**

Page 2. China Kadoorie Biobank collaborative group and members

Page 3. eTable 1: ICD-10 codes for causes of death

Page 4. eTable 2: Number of events by diabetes status at baseline

Page 5. eFigure 1. Association of baseline BMI with cause-specific CVD mortality among individuals with and without diabetes

Page 6. eFigure 2. Association of baseline BMI with cause-specific non-CVD mortality among individuals with and without diabetes

Page 7. eFigure 3: Association of baseline BMI with immediate case fatality and all-cause mortality post non-fatal CVD, among individuals with and without diabetes A) in never-regular smokers and B) after excluding first 5 years of follow-up

Page 8. eFigure 4: Associations of baseline BMI (per 1 kg/m$^2$ higher) at BMI <25 kg/m$^2$ with mortality following CVD events among individuals with and without diabetes, applying various exclusions

Page 9. eFigure 5. Association of baseline BMI with CVD incidence and immediate and long term mortality post non-fatal CVD event among individuals with self-reported and screen detected diabetes

Page 10. eFigure 6. Association of baseline BMI with i) CVD and ii) non-CVD mortality among individuals with and without diabetes by sex

Page 11. eFigure 7: Association of baseline BMI with i) CVD, ii) non-CVD, and iii) all-cause mortality among individuals with and without diabetes by age at baseline

Page 12. eFigure 8. Association of baseline BMI with i) CVD and ii) non-CVD mortality among individuals with and without diabetes in urban and rural areas

Page 13. eFigure 9. Association of sex-specific quintiles of baseline BMI with CVD incidence, immediate case fatality and all-cause mortality post non-fatal CVD event, among individuals with and without diabetes

Page 14. eFigure 10. Association of baseline body fat percentage, lean body mass and fat body mass with CVD incidence, immediate case fatality and all-cause mortality post non-fatal CVD event, among individuals with and without diabetes

## China Kadoorie Biobank collaborative group and members

**International Steering Committee:** Junshi Chen, Zhengming Chen (PI), Robert Clarke, Rory Collins, Yu Guo, Liming Li (PI), Chen Wang, Jun Lv, Richard Peto, Robin Walters.

**International Co-ordinating Centre, Oxford:** Daniel Avery, Derrick Bennett, Ruth Boxall, Fiona Bragg, Ka Hung Chan, Yumei Chang, Yiping Chen, Zhengming Chen, Robert Clarke, Huaidong Du, Zammy Fairhurst-Hunter, Wei Gan, Simon Gilbert, Alex Hacker, Parisa Hariri, Mike Hill, Michael Holmes, Pek Kei Im, Andri Iona, Maria Kakkoura, Christiana Kartsonaki, Rene Kerosi, Om Kurmi, Kuang Lin, John McDonnell, Iona Millwood, Qunhua Nie, Alfred Pozarickij, Paul Ryder, Sam Sansome, Dan Schmidt, Paul Sherliker, Rajani Sohoni, Becky Stevens, Iain Turnbull, Robin Walters, Lin Wang, Neil Wright, Ling Yang, Xiaoming Yang, Pang Yao.

**National Co-ordinating Centre, Beijing:** Yu Guo, Xiao Han, Can Hou, Chun Li, Chao Liu, Jun Lv, Pei Pei, Canqing Yu.

**10 Regional Co-ordinating Centres:**

**Guangxi** Provincial CDC: Naying Chen, Duo Liu, Zhenzhu Tang. Liuzhou CDC: Ningyu Chen, Qilian Jiang, Jian Lan, Mingqiang Li, Yun Liu, Fanwen Meng, Jinhuai Meng, Rong Pan, Yulu Qin, Ping Wang, Sisi Wang, Liuping Wei, Liyuan Zhou. **Gansu** Provincial CDC: Caixia Dong, Pengfei Ge, Xiaolan Ren. Maiji CDC: Zhongxiao Li, Enke Mao, Tao Wang, Hui Zhang, Xi Zhang. **Hainan** Provincial CDC: Jinyan Chen, Ximin Hu, Xiaohuan Wang. Meilan CDC: Zhendong Guo, Huimei Li, Yilei Li, Min Weng, Shukuan Wu. **Heilongjiang** Provincial CDC: Shichun Yan, Mingyuan Zou, Xue Zhou. Nangang CDC: Ziyan Guo, Quan Kang, Yanjie Li, Bo Yu, Qinai Xu. **Henan** Provincial CDC: Liang Chang, Lei Fan, Shixian Feng, Ding Zhang, Gang Zhou. Huixian CDC: Yulian Gao, Tianyou He, Pan He, Chen Hu, Huarong Sun, Xukui Zhang. **Hunan** Provincial CDC: Biyun Chen, Zhongxi Fu, Yuelong Huang, Huilin Liu, Qiaohua Xu, Li Yin. Liuyang CDC: Huajun Long, Xin Xu, Hao Zhang, Libo Zhang. **Jiangsu** Provincial CDC: Jian Su, Ran Tao, Ming Wu, Jie Yang, Jinyi Zhou, Yonglin Zhou. Suzhou CDC: Yihe Hu, Yujie Hua, Jianrong Jin Fang Liu, Jingchao Liu, Yan Lu, Liangcai Ma, Aiyu Tang, Jun Zhang. **Qingdao** Qingdao CDC: Liang Cheng, Ranran Du, Ruqin Gao, Feifei Li, Shanpeng Li, Yongmei Liu, Feng Ning, Zengchang Pang, Xiaohui Sun, Xiaocao Tian, Shaojie Wang, Yaoming Zhai, Hua Zhang, Licang CDC: Wei Hou, Silu Lv, Junzheng Wang. **Sichuan** Provincial CDC: Xiaoyu Chang, Xiaofang Chen, Xianping Wu, Ningmei Zhang. Pengzhou CDC: Xiaofang Chen, Jianguo Li, Jiaqiu Liu, Guojin Luo, Qiang Sun, Xunfu Zhong. **Zhejiang** Provincial CDC: Weiwei Gong, Ruying Hu, Hao Wang, Meng Wan, Min Yu. Tongxiang CDC: Lingli Chen, Qijun Gu, Dongxia Pan, Chunmei Wang, Kaixu Xie, Xiaoyi Zhang.

## eTable 1. ICD-10 codes for causes of death

| Cause of death | ICD-10 codes |
|---|---|
| **CVD** | I00-I99 |
| **IHD** | I20-I25 |
| **Stroke** | I60-I61, I63-I64 |
| **IS** | I63 |
| **ICH** | I61 |
| **Non-CVD** | |
| **COPD** | I26-I27, J41-J44 |
| **Cancer** | C00-C97 |
| **Diabetic ketoacidosis or coma** | E10.0, E11.0, E12.0, E13.0, E14.0, E10.1, E11.1, E12.1, E13.1, E14.1 |
| **Kidney disease** | N02-N03, N07, N11 N18 |

COPD= chronic obstructive pulmonary disease, CVD=cardiovascular disease, ICH= intracerebral haemorrhage, IHD=ischaemic heart disease, IS=ischaemic stroke

## eTable 2. Number of events by diabetes status at baseline

| Cause of death or event | No. of events (%) | |
|---|---|---|
| | No diabetes (n=422,871) | Diabetes (n=23,842) |
| **Deaths from any cause** | 23,305 (5.5) | 3509 (14.7) |
| **Non-CVD deaths** | 15,011 (3.5) | 2078 (8.7) |
| **COPD** | 932 (0.2) | 81 (0.3) |
| **Cancer** | 9032 (2.1) | 823 (3.5) |
| **Diabetic ketoacidosis or coma** | 26 (0.01) | 139 (0.6) |
| **Kidney disease** | 339 (0.08) | 232 (1.0) |
| **CVD deaths** | 8294 (2.0) | 1431 (6.0) |
| **IHD** | 2862 (0.7) | 592 (2.5) |
| **Stroke** | 3983 (0.9) | 594 (2.5) |
| **IS** | 828 (0.2) | 188 (0.8) |
| **ICH** | 2920 (0.7) | 381 (1.6) |
| **All CVD events** | 99,122 (23.4) | 9943 (41.7) |
| **Survived to the end of follow-up** | 86,800 (20.5) | 7664 (32.1) |
| **Immediate case fatality (died within 28 days)** | 4693 (1.1) | 671 (3.0) |
| **All-cause mortality post non-fatal CVD event** | 6773 (1.6) | 1474 (6.1) |
| **CVD mortality post non-fatal CVD event** | 3601 (0.9) | 760 (3.2) |

COPD= chronic obstructive pulmonary disease, CVD=cardiovascular disease, ICH= intracerebral haemorrhage, IHD=ischaemic heart disease, IS=ischaemic stroke

## eFigure 1. Association of baseline BMI with cause-specific CVD mortality among individuals with and without diabetes

Hazard ratios (HRs) are stratified by age-at-risk, sex and study area, and adjusted for education, smoking, alcohol and physical activity. HRs are plotted on a floating absolute risk scale and separate y-axis scales were used for individuals with and without diabetes (black and grey labels, respectively). HRs are relative to 22.5-24.9 kg/m$^2$ group, separately in individuals with and without diabetes. Each closed square represents HR with the area inversely proportional to the variance of the log HR. Vertical lines indicate 95% CIs. The $\bar{x}$ above the x-axis represents the mean value of BMI in the full CKB population and the ±1S and ±2S represent 1 and 2 SD from the mean, respectively.

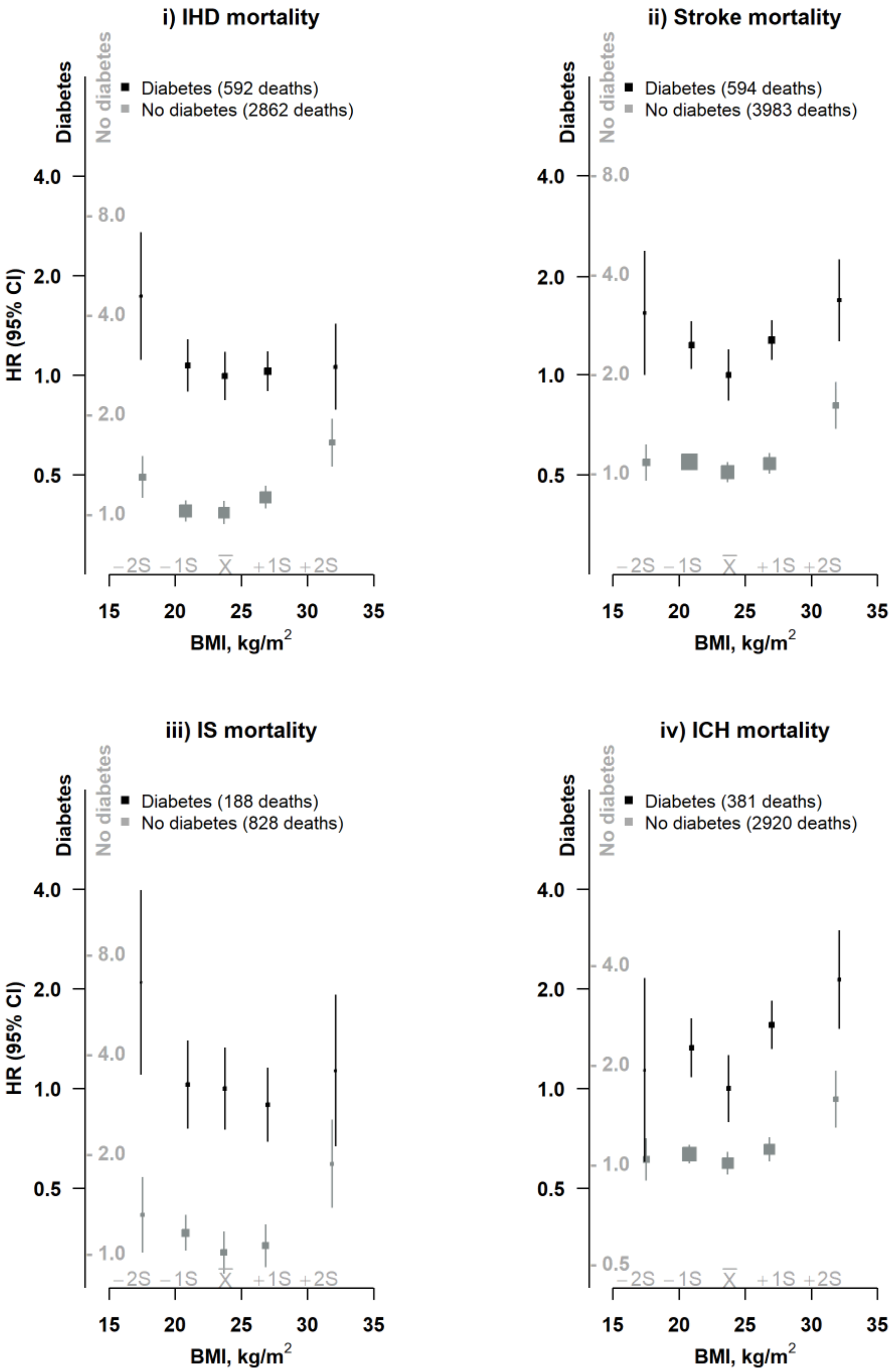

**eFigure 2. Association of baseline BMI with cause-specific non-CVD mortality among individuals with and without diabetes**

Conventions as eFigure 1.

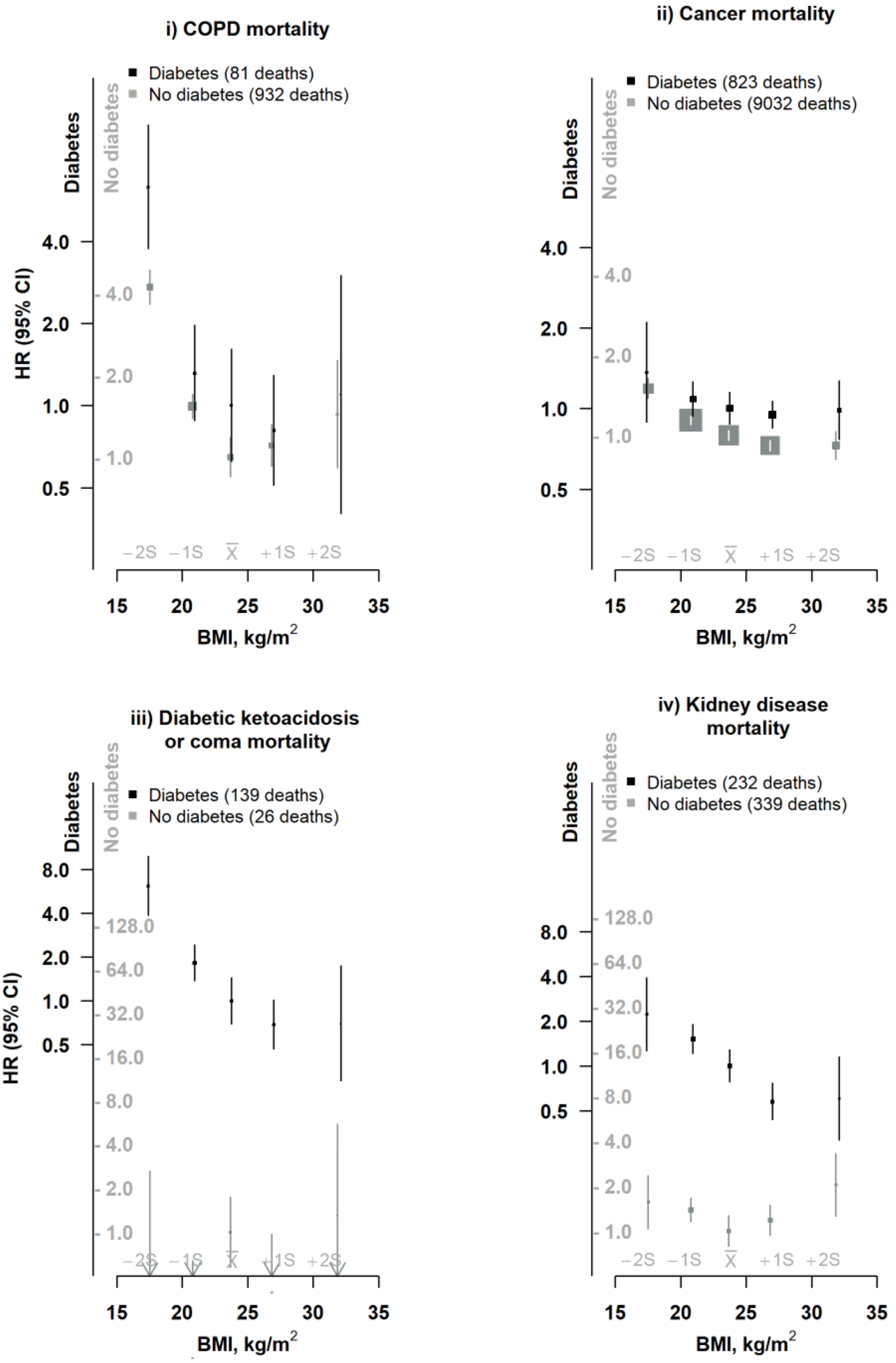

**eFigure 3. Association of baseline BMI with immediate case fatality and all-cause mortality post non-fatal CVD, among individuals with and without diabetes A) in never-regular smokers and B) after excluding first 5 years of follow-up**

Conventions as eFigure 1.

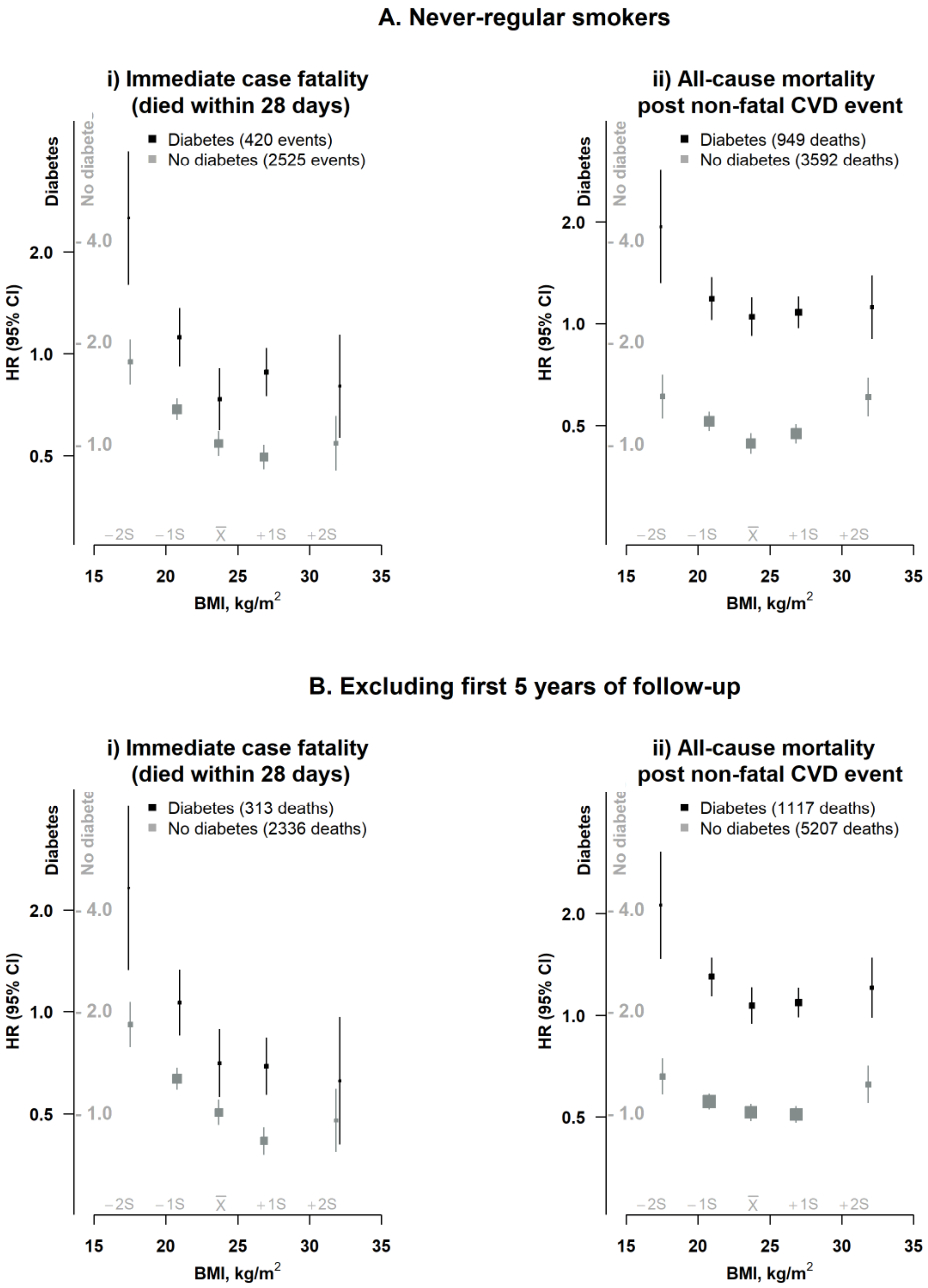

**eFigure 4. Associations of baseline BMI (per 1 kg/m$^2$ higher) at BMI <25 kg/m$^2$ with mortality following CVD events among individuals with and without diabetes, applying various exclusions**

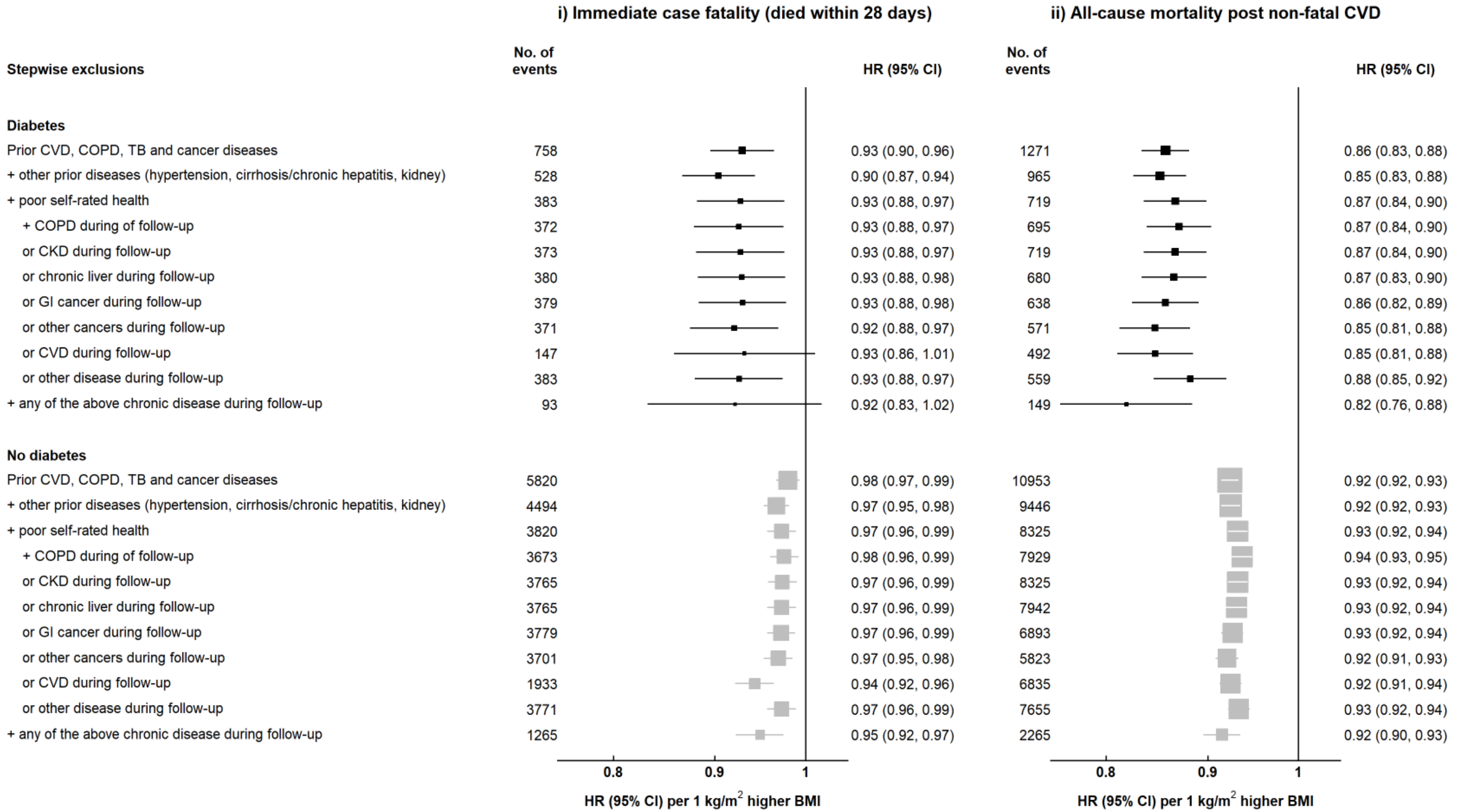

**eFigure 5. Association of baseline BMI with CVD incidence and immediate and long term mortality post non-fatal CVD event among individuals with self-reported and screen detected diabetes**

Conventions as eFigure 1.

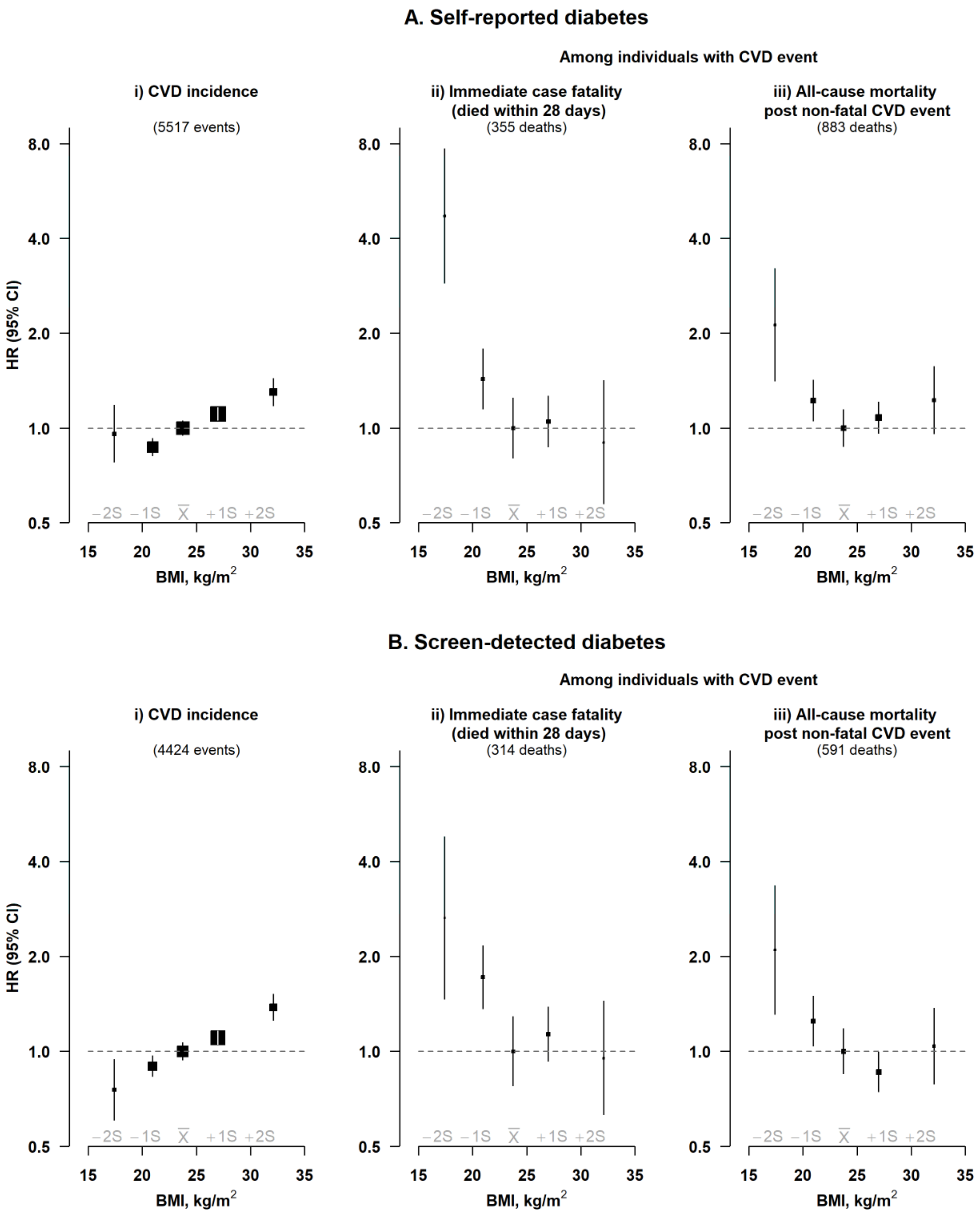

**eFigure 6. Association of baseline BMI with i) CVD and ii) non-CVD mortality among individuals with and without diabetes by sex**

Conventions as eFigure 1.

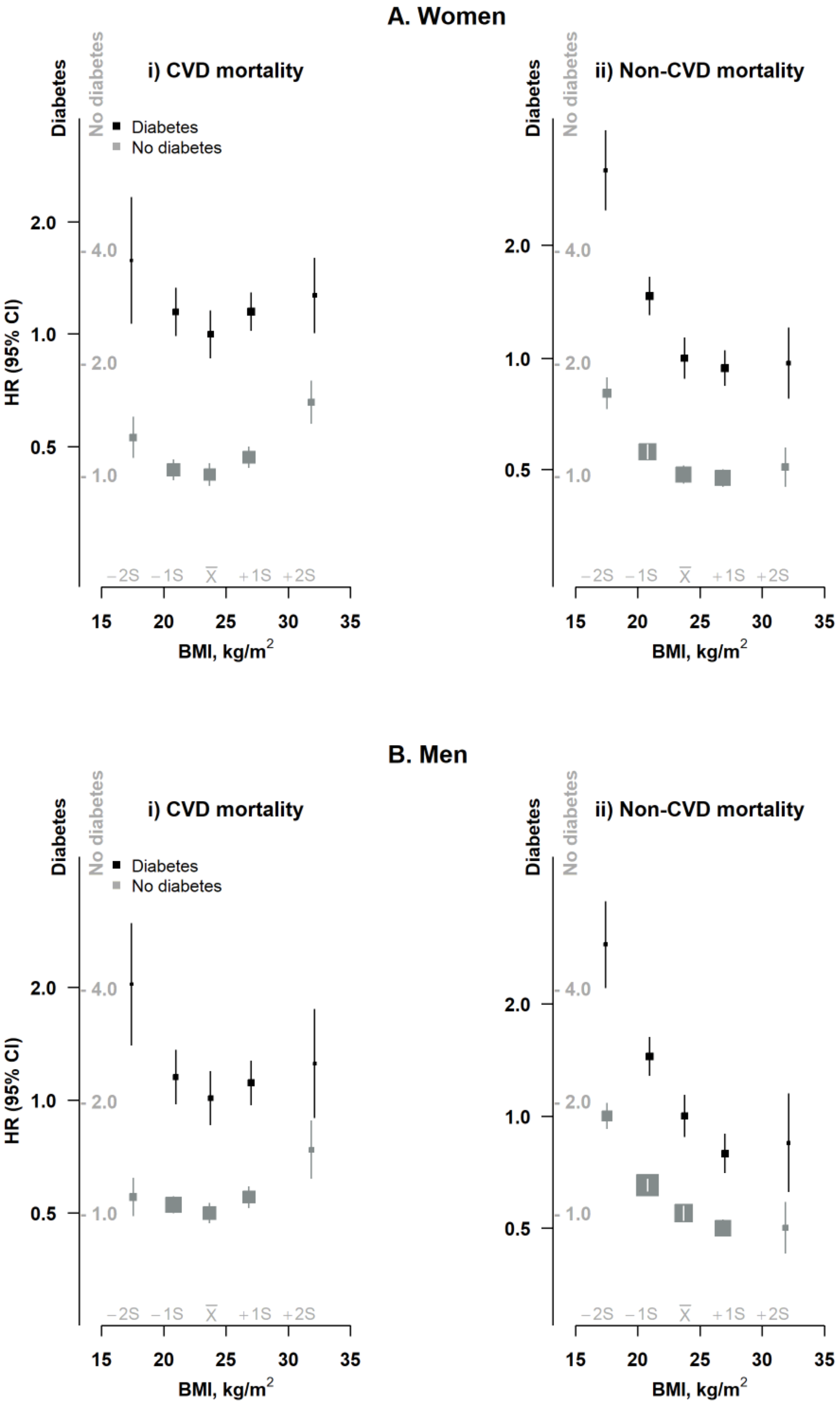




Iona A, *et al. BMJ Open Diab Res Care* 2022; 10:e002489. doi: 10.1136/bmjdrc-2021-002489

**eFigure 7. Association of baseline BMI with i) CVD, ii) non-CVD, and iii) all-cause mortality among individuals with and without diabetes by age at baseline**

Conventions as eFigure 1.

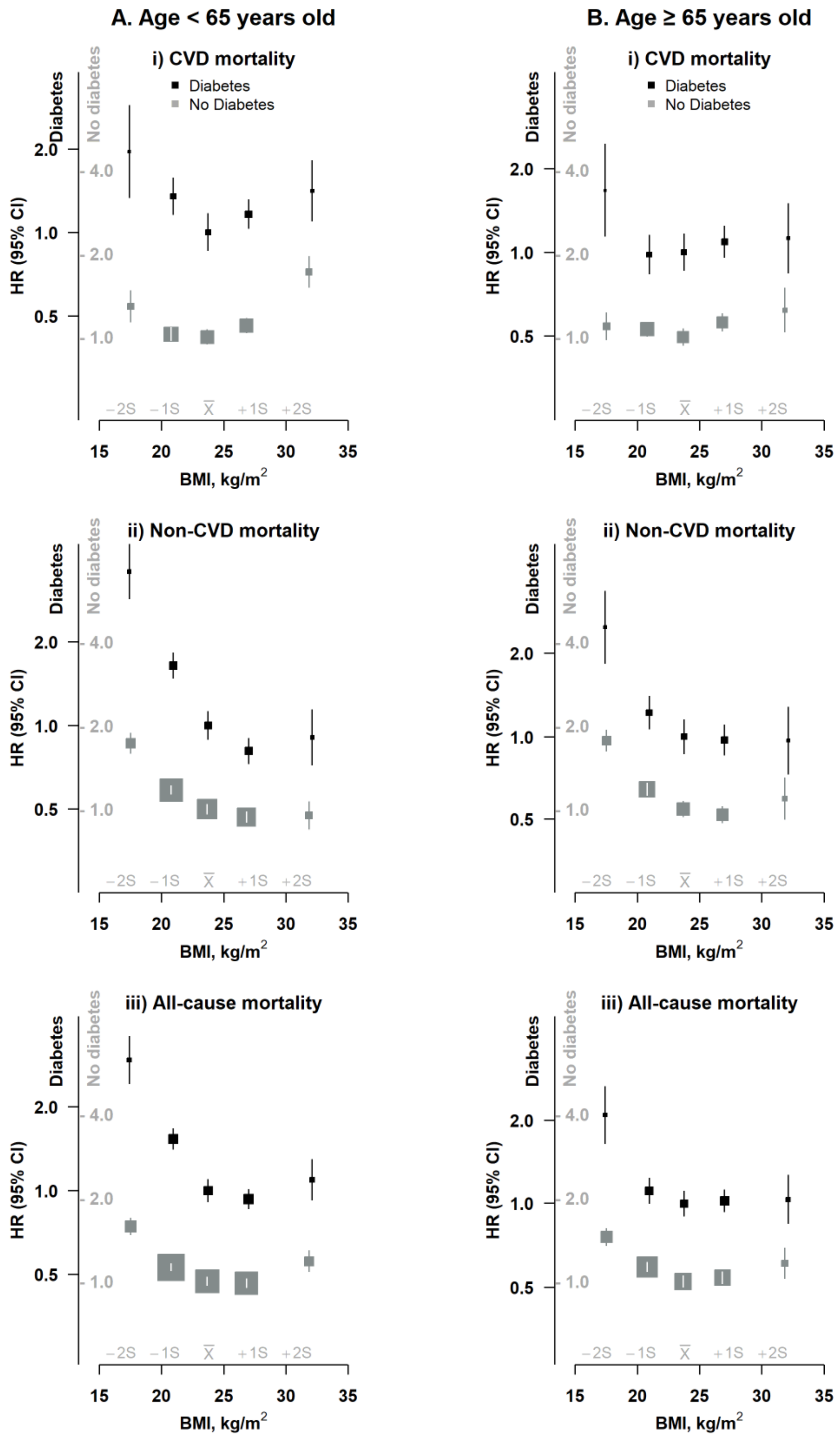

## eFigure 8. Association of baseline BMI with i) CVD and ii) non-CVD mortality among individuals with and without diabetes in urban and rural areas

Conventions as eFigure 1.

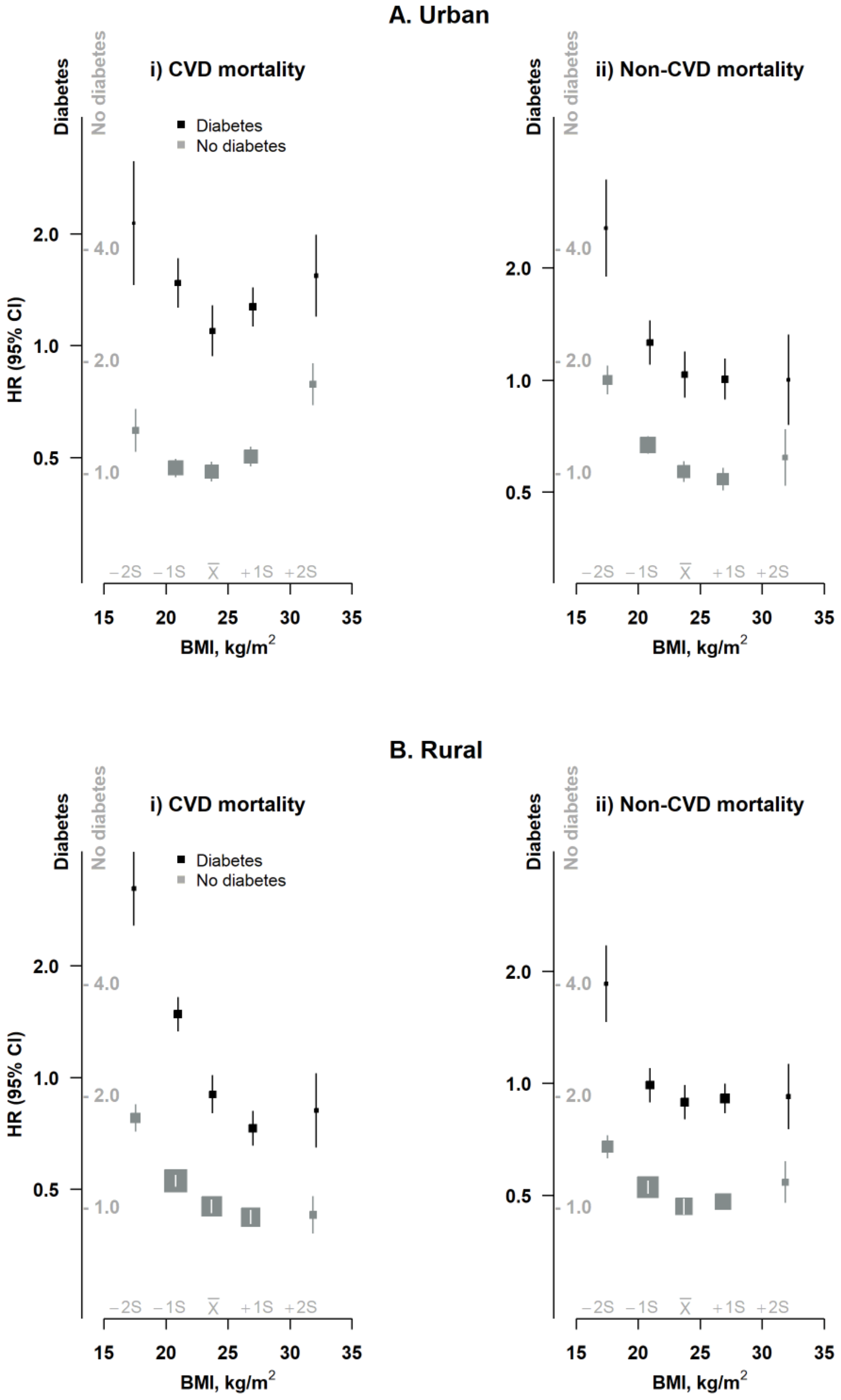

**eFigure 9. Association of sex-specific quintiles of baseline BMI with CVD incidence, immediate case fatality and all-cause mortality post non-fatal CVD event, among individuals with and without diabetes**

Hazard ratios (HRs) are stratified by age-at-risk, sex and study area, and adjusted for education, smoking, alcohol and physical activity. HRs are plotted on a floating absolute risk scale and separate y-axis scales were used for individuals with and without diabetes (black and grey labels, respectively). HRs are relative to 3rd quintile group, separately in individuals with and without diabetes. Each closed square represents HR with the area inversely proportional to the variance of the log HR. Vertical lines indicate 95% CIs. The $\bar{x}$ above the x-axis represents the mean value of each adiposity measure in the full CKB population and the ±1S and ±2S represent 1 and 2 SD from the mean, respectively.

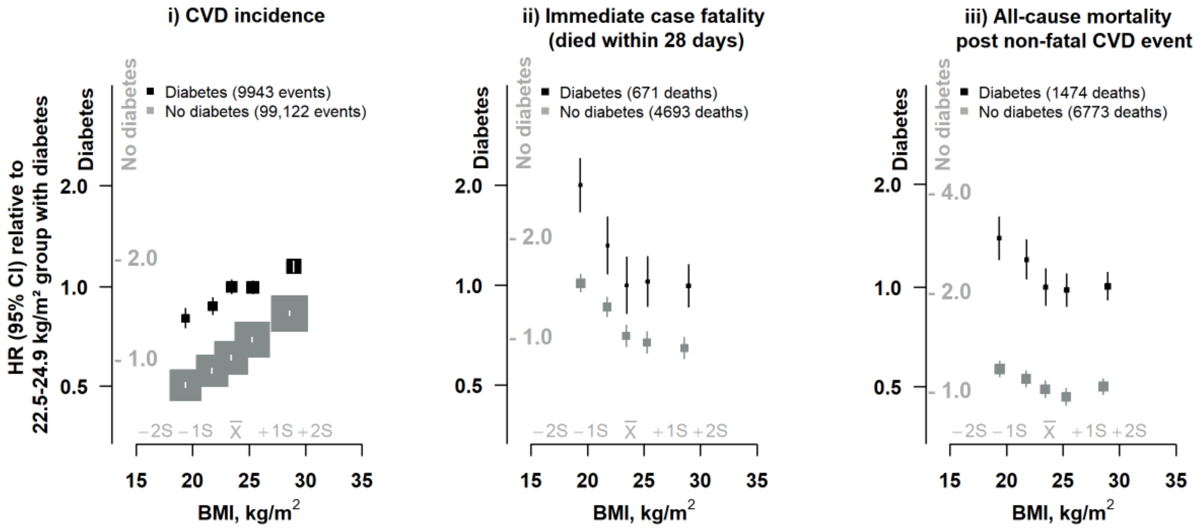




Iona A, et al. BMJ Open Diab Res Care 2022; 10:e002489. doi: 10.1136/bmjdrc-2021-002489

**eFigure 10. Association of baseline body fat percentage, lean body mass and fat body mass with CVD incidence, immediate case fatality and all-cause mortality post non-fatal CVD event, among individuals with and without diabetes**

Conventions as eFigure 9.

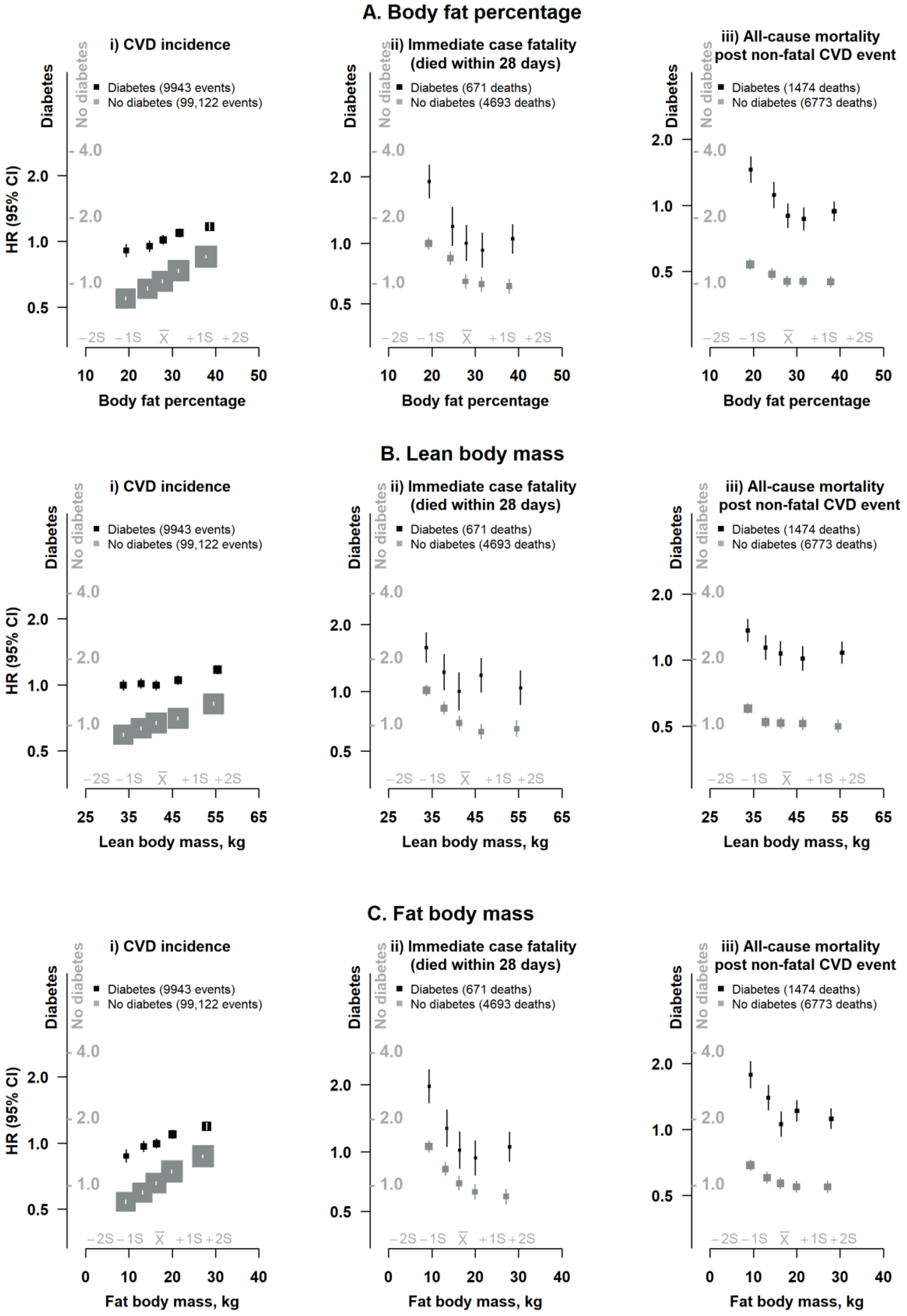




Iona A, *et al. BMJ Open Diab Res Care* 2022; 10:e002489. doi: 10.1136/bmjdrc-2021-002489
